# Hemostatic Effects of Microbubble-Enhanced Low-Intensity Ultrasound in a Liver Avulsion Injury Model

**DOI:** 10.1371/journal.pone.0095589

**Published:** 2014-05-01

**Authors:** Guiying Feng, Jianhua Liu, Xiaochen Zhao, Jinglu Wei, Wencai Ou, Shuyi Xiao, Zhiwen Hu, Hongqin Wei, Zheng Liu

**Affiliations:** 1 Department of Medical Ultrasound, Guangzhou First People’s Hospital, Guangzhou Medical University, Guangzhou, China; 2 Department of Ultrasound, Xinqiao Hospital, the Third Military Medical University, Chongqing, China; NIH, United States of America

## Abstract

Microbubble-enhanced therapeutic ultrasound (MEUS) can block the blood flow in the organs. The aim of this study was to evaluate the hemostatic effect of microbubble-enhanced pulsed, low-intensity ultrasound in a New Zealand White rabbit model of avulsion trauma of the liver. The therapeutic ultrasound (TUS) transducer was operated with the frequency of 1.2 MHz and an acoustic pressure of 3.4 MPa. Microbubble-(MB) enhanced ultrasound (MEUS) (n = 6) was delivered to the distal part of the liver where the avulsion was created. Livers were treated by TUS only (n = 4) or MB only (n = 4) which served as controls. Bleeding rates were measured and contrast enhanced ultrasound (CEUS) was performed to assess the hemostatic effect, and liver hemoperfusion before and after treatment. Generally, bleeding rates decreased more than 10-fold after the treatment with MEUS compared with those of the control group (P<0.05). CEUS showed significant declines in perfusion. The peak intensity value and the area under the curve also decreased after insonation compared with those of the control group (P<0.05). Histological examination showed cloudy and swollen hepatocytes, dilated hepatic sinusoids, perisinusoidal spaces with erythrocyte accumulation in small blood vessels, obvious hemorrhage around portal areas and scattered necrosis in liver tissues within the insonation area of MEUS Group. In addition, necrosis was found in liver tissue 48 h after insonation. We conclude that MEUS might provide an effective hemostatic therapy for serious organ trauma such as liver avulsion injury.

## Introduction

The liver is a fragile structure with a rich blood supply provided by the hepatic artery and the portal vein. Due to its large size and relatively fixed location, it is rather susceptible to traumatic injury. According to the literature, liver trauma accounts for about 15%–20% of all the types of abdominal trauma in humans [1]. There are currently several emergency treatment approaches to liver rupture including gauze packing, electric coagulation, argon hemostasis, simple suture, hepatic artery embolization or ligation, and partial hepatectomy [2]. Surgical approaches are effective, but can also result in operative damage[3]–[5]. Selective hepatic artery ligation can lead to the necrosis of soft tissue, vascular injury and bleeding as well as the leakage of intrahepatic bile duct [6]. The American Association for the Surgery of Trauma (AAST) has proposed that patients with scores less III for liver trauma, and with stable blood circulation might be treated by non-surgical approaches [7].

Minimally invasive methods guided by contrast enhanced ultrasound(CEUS)to stop bleeding include hemostatic agent injection, percutaneous radiofrequency coagulation, microwave coagulation and high-intensity focused ultrasound (HIFU) [8]–[16]. These methods can result in hemostatic effects, less invasiveness, and rapid recovery[8]–[16]. However, the efficacy of the hemostatic agents injected in cases of large hemorrhage due to serious trauma have not been satisfactory, and can cause pain [8]–[10]. The principle of percutaneous radiofrequency coagulation, microwave coagulation, and high-intensity focused ultrasound (HIFU) is to cause hemostasis by heating the target tissue to cause coagulation necrosis[11]–[16]. Such methods have certain limitations. For example, temperatures above 70°C often lead to dehydration and atrophy of the tissue with deformation of the adjacent structures [11]. However, if the blood flow in the target area is high, the heat can be dissipated during the application process (heat sink effect), which can permit results in prolonged treatment [15]. In addition, the above hemostatic methods can only work with direct contact applied to a point or limited a surface area.

Acoustic hemostasis refers to a non-invasive method which takes advantage of the thermal and cavitation effect of ultrasound in order to reduce bleeding. Experiments on animals have shown that the use of HIFU resulted in resolution of massive bleeding caused by tissue cutting of the liver, spleen, and in injury of blood vessels of 2–10 mm in diameter [11]–[14]. However, due to the heat sink effect [15] and the narrow area of direct therapeutic coagulation by HIFU, achieving hemostasis over large areas is difficult. Studies have shown that cavitation by HIFU treatment could result in the formation of micro-thrombosis within small blood vessels, and production of homogenates over the wound surface. This prevents the wound from bleeding, promotes the absorption of ultrasonic energy to increase the regional temperature above 80°C, and enhances thermal hemostasis [17], [18].

Microbubbles (MB) can not only sensitively identify the hemorrhagic location and monitor the progress of the procedures, but it also can be used to block blood flow in the organs by damaging tiny blood vessels, when combined with therapeutic ultrasound, namely by angiotripsy [19], [20]. In our previous studies on organ trauma in animals such as dogs and rabbits, it was demonstrated that cavitation could block the microcirculation, and control bleeding of livers and spleens in a laceration model [21], [22]. However, the incisions made in those trauma models were neat cuts corresponding to injury scale I [7]. In that model, the hemorrhage was relatively small and unstable. Hemostasis could be achieved by simple compression of the cut surfaces. Therefore, factors such as activation of clotting and contact compression causing observed hemostasis could not be excluded.

The aim of this study was to further explore the feasibility, effectiveness and histological characteristics of microbubble-enhanced ultrasound (MEUS) hemostasis in a rabbit model of another type of liver trauma, liver avulsion injury (injury scale II [7]).

## Materials and Methods

### Ethics Statement

All procedures were reviewed and approved by the Animal Care Committee of the Guangzhou First People’s Hospital, Guangzhou Medical University. All animals were anesthetized by intravenous injection of 2% pentobarbital at a dose of 1.5 ml/kg. The room temperature was kept at about 25°C, and the animals were maintained under deep anesthesia during the experiments.

### Animal Preparation

Fourteen healthy male New Zealand White rabbits weighing about 2.0–2.5 kg were obtained from the Guangdong Medical Laboratory Animal Center. All rabbits were fasted for 12 h before surgery, but had free access to water. They were randomly assigned to three groups: microbubble-enhanced ultrasound (MEUS) (n = 6), therapeutic ultrasound alone without microbubbles (TUS) (n = 4), and microbubbles only with sham ultrasound exposure (MB) (n = 4).

### Pulsed Therapeutic Ultrasound Device

The device (DCT-700) was Security type Class I, Type B (Shenzhen Well.D Medical Electronics Co. Ltd. Shenzhen, China). The acoustic pressure power was AC100V–240V with a continuous modulated waveform and pulse set on optional. The ultrasound transducer was 2.3 cm in diameter composed of an aluminum shell with a tip covered with a polyimide membrane. A needle hydrophone (TNU001A, NTR, Seattle, WA, USA) was positioned to measure the acoustic output at a depth of 0.5 cm from the surface. The transducer was operated at 1.2 MHz with a pulse repetition of 10 Hz, and an actual acoustic pressure is 3.4 Mpa while the stalls in one was 2000 Kpa. The intermittent mode of transducer was 6 s on and 6 s off and the actual working duty was 0.15% with an insonation time of 5 min. The spatial peak temporal average intensity (I_SPTA_) was 1.182 W/cm^2^. The insonation procedure was performed by the same experimenter for all animals.

### Microbubble Contrast Agents

Zhifuxian [23], a second generation contrast agent, was produced by the Department of Ultrasound, Xinqiao Hospital, The Third Military Medical University, Chongqing, China. The microbubbles could be used not only for CEUS, but also for nucleation for ultrasound cavitation. The liposome membrane of Zhifuxian was composed of dipalmitoyl phosphatidylglycerol (DPPG), distearoyl phosphatidylcholine (DSPC), and polyethylene glycol-4000 (PEG-4000), and the core gas was perfluoropropane. The mean microbubble diameter was 2.3 cm, and the concentration was 4–9×10^9^/ml. Microbubbles were oscillated for 45 s, and then injected in an ear vein at doses of 0.02 ml/kg for CEUS, and 0.1 ml/kg for insonation.

### Precision Scale

A precision scale (Sartorius BS 300S-WEL) (Beijing Sartorius Balance Co., Beijing, China) was used which had a lower limit of measurement of 0.001 g, and a maximum limit of 300 g. The balance was zeroed before each use.

### Experimental Procedure

**Figure 1:**
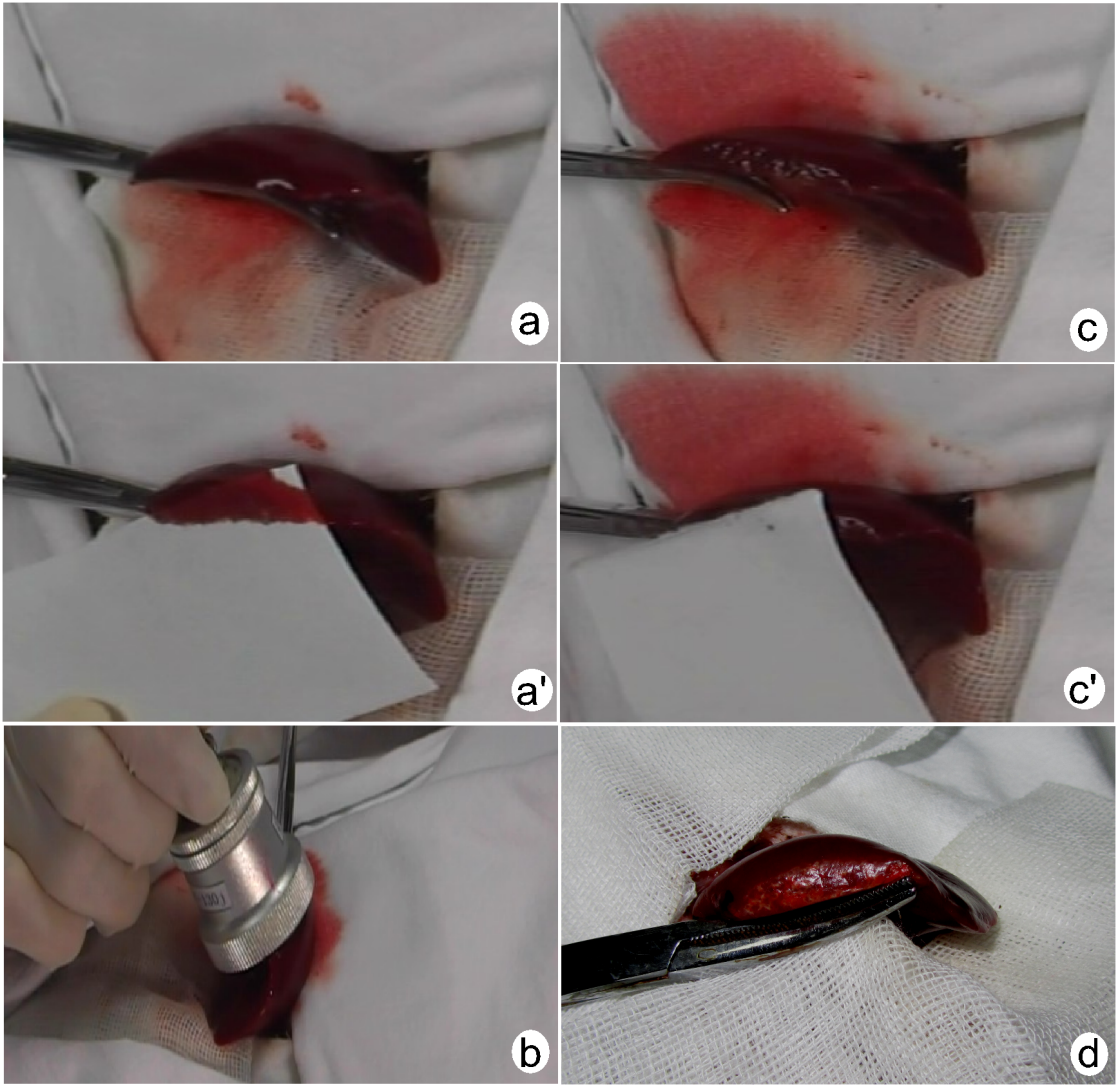
Images of procedure for application of microbubble-enhanced therapeutic ultrasound. **a**’, active bleeding of the wound before insonation; **b**, Direct insonation of the wound by a transducer during injection of microbubbles; **c**, **c’,** small exudates over the wound after insonation; **d**, The liver wound were smooth and no bleeding after 48 h.

Anesthetized animals placed in a supine position, and the fur of the upper anterior abdomen was removed. The abdomen was opened through a midline incision and the left hepatic lobe was stretched out carefully with saline gauze, then fixed in situ. A model of a liver avulsion wound 2.7 cm in length and 1.5 cm in depth was created about 0.8 cm from the edge of the liver using a scissor. The blood from wound was collected with absorbent paper for 30 s, and weighed on the precision scale. Then, the wound was insonated vertically and directly with the transducer (Fig. 1). For the MEUS group, the wound bed was insonated directly for 5 min with intravenous injection of microbubbles (0.1 ml/kg, diluted with saline to 2 ml) in the first 4 min. The treatment transducer moved at uniform speed without any pressure to insonate throughout the wound. For the TUS group, the same method was used to insonate the wound, but 2 ml saline solution instead of microbubbles was injected intravenously. For the MB Group, the same dose of microbubbles was injected intravenously in the first 4 min, and in the same way to insonate the wound, but no pulses were transmitted. After insonation, clot over the liver wound was removed, and blood was collected again for 30 s. After the experiment, we randomly selected three rabbits from the MEUS group, and all rabbits from the control groups were euthanized by intravenous injection of pentobarbital at a dose of 100 mg/kg. Tissue samples were obtained from the midpoint of the wound for histological examination. The abdominal wall of the remaining 3 rabbits was closed after the animals were fed for 48 h, and were given intramuscular injections of penicillin and gentamicin daily to prevent infection. The remainder of the animals were euthanized and prepared for histological examination in the same way. These samples were fixed in 10% formalin, embedded in paraffin and stained with hematoxylin and eosin (HE).

### Hemostatic Effect Analysis

Visual scores of bleeding were recording as described previously [22], using the scoring criteria: complete hemostasis, grade 0; slow and small amount of exudates, grade 1; blood slowly oozing and minor activity, grade 2; profuse activity bleeding, grade 3; significantly profuse bleeding, grade 4. The visual scores of hemostatic effects were evaluated by the same operators who were blind to the study process.

Quantitative analysis of the hemostatic effect was performed by comparing the bleeding rate before and after insonation among all groups. The clean absorbent papers were weighed by a precision scale for dry weight, and again after 30 s of absorption of blood for wet weight. The bleeding rate was defined as the difference between the wet and dry weights divided by 30 s.

### CEUS Analysis

A diagnostic ultrasound system (ACUSON S2000, Siemens Medical Solutions USA, Inc. Mountain View, CA, USA), equipped with a 9L4 high-frequency linear array probe (frequency ranged from 4–9 MHz) was used with ultrasound imaging modes, and analyzed by acoustic density analysis software. Depth, gain and other settings remained the same, with a mechanical index (MI) of 0.06, MIF of 0.05, depth of display 3 cm, dynamic range of 80 dB, and a gain of 0 dB during the CEUS studies. For CEUS examination, all rabbits were given an intravenous bolus of microbubbles injected at a concentration of 0.02 ml/kg and in a volume of 2 ml saline. Data were video recorded for 150 s. The models of hepatic trauma were not prepared until 30 minutes after the microbubbles from pre-insonation CEUS examination had disappeared. The perfusion was evaluated by visual observation, and the acoustic density analysis software. The visual evaluation standard of hepatic perfusion for CEUS was: Grade 0, obviously homogeneous enhanced without any negative development; Grade I, medium and large blood vessels enhanced well, but with mild negative development; Grade II, medium and large blood vessels enhanced, but with parenchymal negative development; Grade III, the blood vessels and parenchyma had no real perfusion. All visual evaluations were made by the same operators who were blind to the study protocol. The acoustic density analysis software was employed to analyze the time intensity curve (TIC) of each time point before, and after insonation of liver parenchyma, to select a similar region-of-interest ROI area (approximately 45 mm^2^, with an amplitude of difference less than 1 mm^2^), and to calculate the peak intensity value (PI) and the area under the curve (AUC).

### Data Analysis

Data on bleeding rate, acoustic density analysis (PI, AUC) of each group before and after insonation were expressed as mean ± SD, and the data of bleeding visual scores of each group before and after insonation were expressed as medians (Q25, Q75) because they did not follow a normal distribution. The differences in the bleeding rate, PI, AUC of each group before and after insonation were analyzed by paired-samples Student’s *t*-test, and the data from each group before and after insonation were compared by using one-way ANOVA and LSD test for analyzing the differences between two groups when the total difference was significant. The visual bleeding scores of each group before and after insonation were compared by the Wilcoxon test and data of each corresponding group was compared by the Kruskal-Wallis H and Mann-Whitney U tests. We used Pearson’s correlation to analyze the correlations between bleeding rate with PI, bleeding rate with AUC, and PI with AUC, and Spearman’s correlation was used to analyze the correlation between bleeding rate with visual score. A P value<0.05 was considered to be statistically significant. All the data were analyzed by using SPSS 13.0 software.

## Results

### Hemostatic Effect Analysis

**Figure 2:**
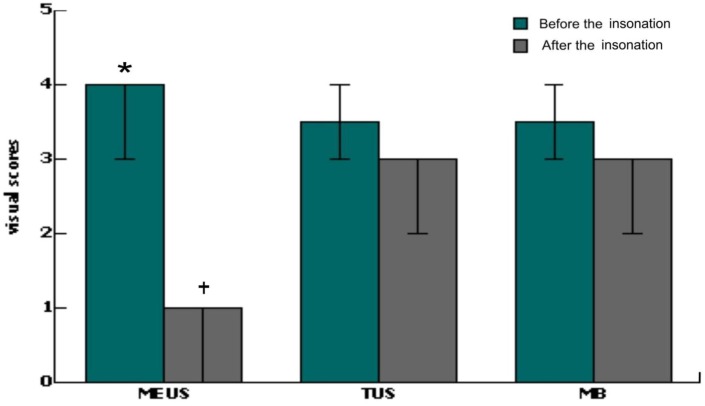
A comparison of bleeding visual scores before and after insonation. *P<0.05 indicates a significant difference between before and after insonation of MEUS group. ^+^P<0.05 indicates a significant difference between MEUS group and the controls.

The bleeding visual score of the MEUS group after the insonation compared with that of pre-insonation situation decreased from grade 4 (3,4) to 1 (0.75,1), and the difference was statistically significant (P<0.05); Differences between the MEUS group and the control group after insonation were statistically significant (P<0.05) (Fig. 1, Fig. 2).

The bleeding rates of livers were similar before treatment, ranging from 0.012±0.007 g/s to 0.019±0.014 g/s. The differences among the three groups were not statistically significant (P>0.05). After insonation, the bleeding rate of the MEUS group decreased from 0.013±0.004 to 0.002±0.001 g/s after insonation (P<0.05). Differences between the MEUS group and the control groups after insonation were significantly different (P<0.05) (Table 1).

**Table 1:** The comparison of bleeding rate before and after insonation (g/s, –x±s).

Groups	Before insonation	After insonation
MEUS	0.013±0.004	0.002±0.001* ^,+^
TUS	0.019±0.014	0.007±0.004
MB	0.012±0.007	0.006±0.002

Data are the means±standard deviations. MEUS means microbubble-enhanced (therapeutic) ultrasound; TUS means therapeutic ultrasound only without microbubbles; MB means no pulses were transmitted by the transducer only injected microbubbles.

*P<0.05 indicates a significant difference between the MEUS groups before and after insonation; and ^+^P<0.05 indicates a significant difference between the MEUS group and the controls.

### Contrast-enhanced Ultrasound

**Figure 3:**
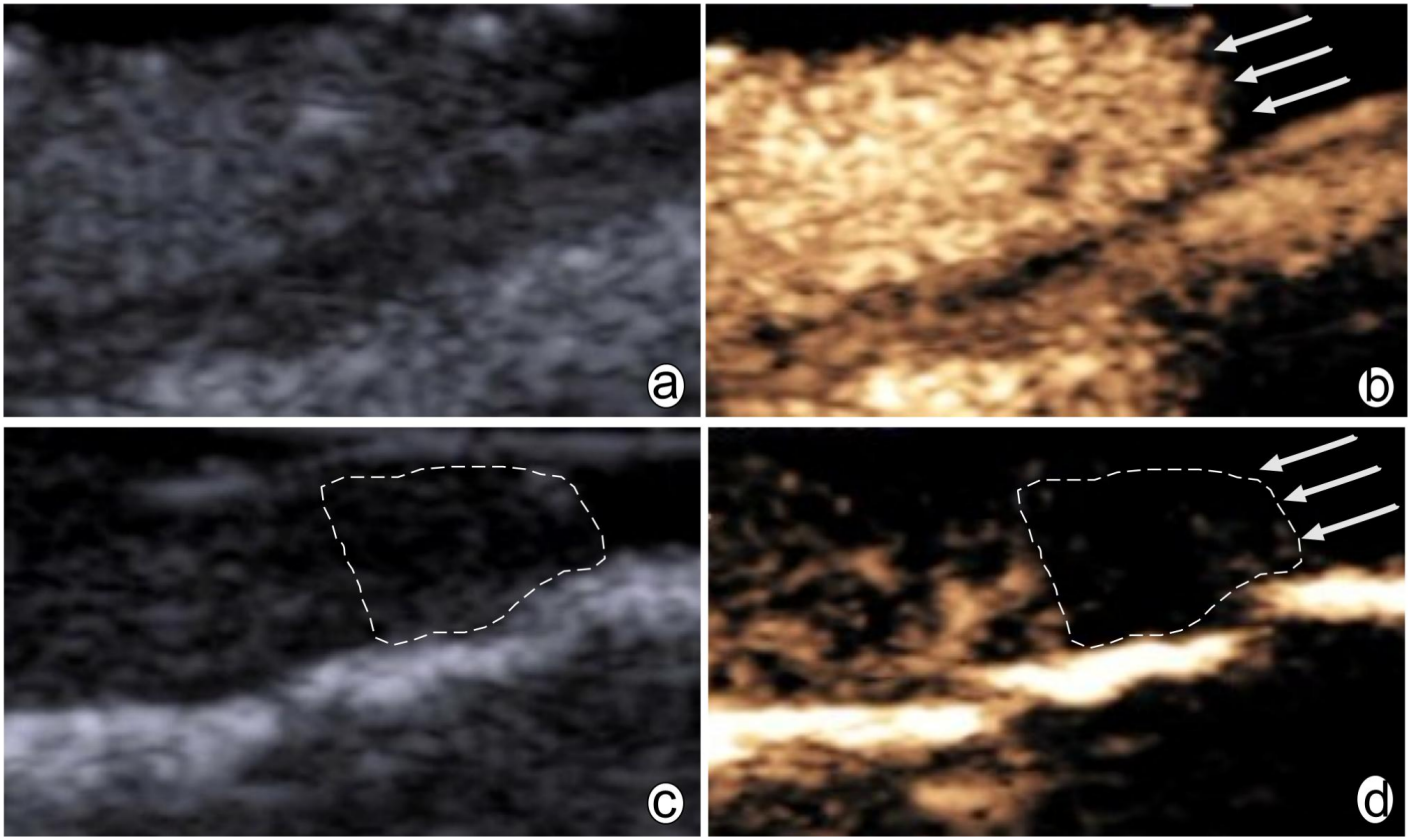
CEUS images after insonation. **a, b**, Uniform perfusion of contrast agent in the MB group; **c, d**, A representative CEUS image showed a perfusion defect (arrow indicates the trauma, breakage the wound, and dotted line shows the range of defects. The area beyond the dotted line also showed an irregular defect) in MEUS group.

Visual evaluation: Contrast agent was well perfused to all the corresponding regions of target livers before treatment, and the insonation regions of TUS group and MB group. Microbubbles arrived at the liver rapidly, and enhanced the tissue homogeneously without any defects. The scoring grade was 0. In the MEUS Group, the microbubbles arrived at liver slowly and unevenly. The liver tissues showed irregular non-enhanced areas of varying sizes with the edges of the defects being poorly perfused. The score was grade II (Fig. 3).

Analysis of acoustic density: Before treatment, the differences in PI and AUC among three groups were not significant (P>0.05). After insonation, the PI of the MEUS Group decreased from 22.000±6.090% to 9.517±1.380%, and AUC decreased from 2173.983±839.190% s to 872.583±196.409% s. The differences were statistically significant (P<0.05). The comparison between the MEUS group and the control groups after insonation were significantly different (P<0.05) (Table 2).

**Table 2:** Comparison of acoustic density analysis before and after insonation(–x±s).

Groups	PI(%)		AUC(%s)	
	Before	After	Before	After
MEUS	22.000±6.090	9.517±1.380* ^,+^	2173.983±839.190	872.583±196.409* ^,+^
TUS	21.325±7.439	20.650±8.925	2021.250±795.858	1960.600±393.361
MB	24.325±7.518	19.900±7.795	2155.125±853.575	1960.725±622.246

Data are the means±standard deviations. MEUS means microbubble-enhanced (therapeutic) ultrasound; TUS means therapeutic ultrasound only without microbubbles; MB means no pulses were transmitted by the transducer only injected microbubbles.

*P<0.05 indicates a significant difference between before and after insonation of the MEUS group. ^+^P<0.05 indicates a significant difference between the MEUS group and the controls.

Correlation analysis: there was excellent positive correlation between bleeding rate, and PI and AUC, after insonation (P<0.05 for both). The correlation of PI and AUC between visual score and bleeding rate was positive (P<0.05) (Table 3).

**Table 3:** Correlation between bleeding rates and acoustic density analysis, bleeding visual score before and after insonation.

	Before insonation		After insonation	
	r	p	r	p
Bleeding rate and PI	0.386	0.173	0.763	0.001
Bleeding rate and AUC	0.250	0.389	0.700	0.005
PI and AUC	0.779	0.001	0.914	0.000
Bleeding rate and Bleeding visual score	–0.251	0.387	0.568	0.034

PI means the peak intensity value, and AUC means the area under the curve.

### Histological Examinations

**Figure 4:**
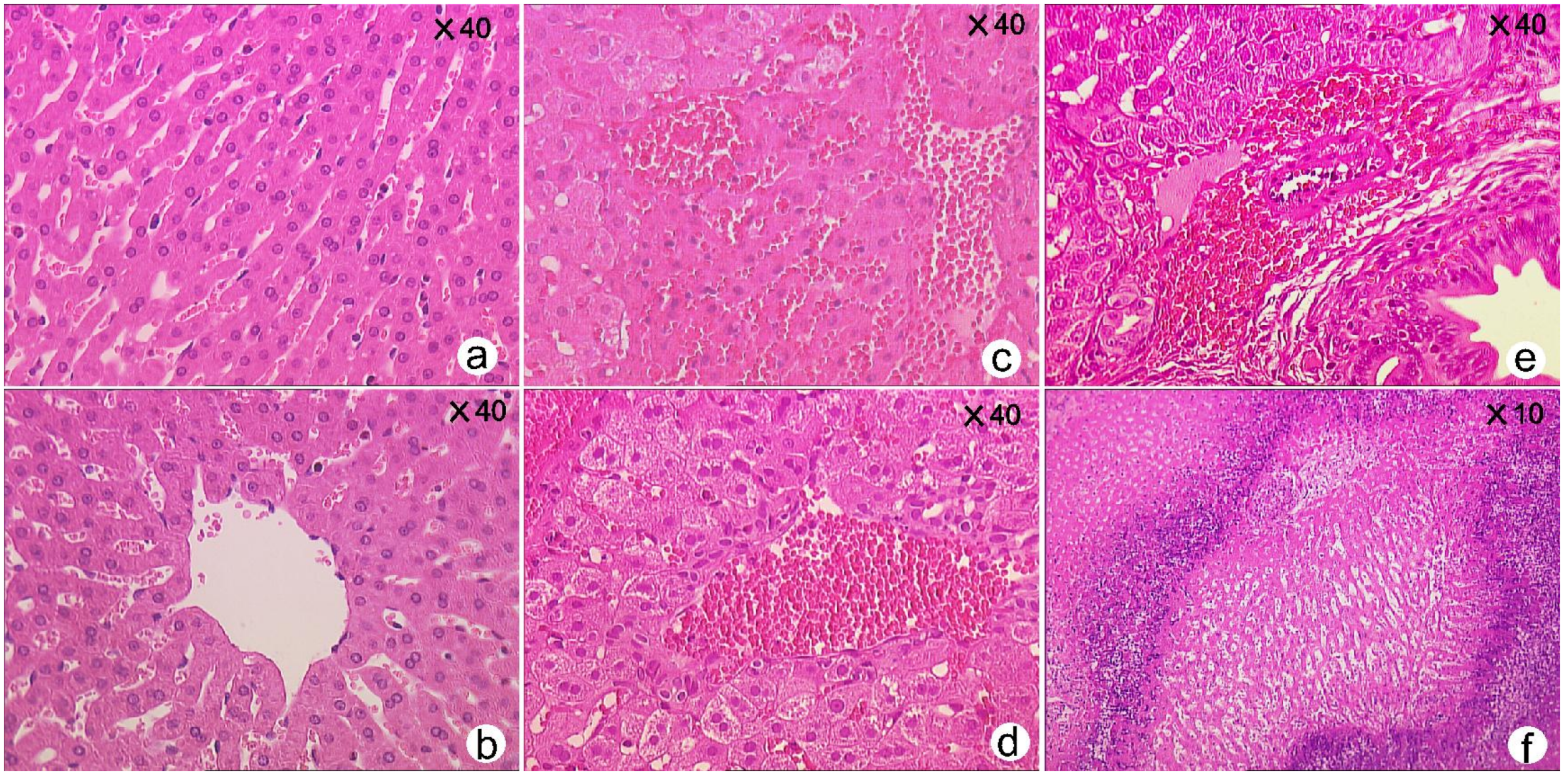
Representative histological HE sections after insonation of the TUS, and, MB groups. Hepatic cord and plate structure were clear, and blood cells were scattered in the sinusoids (**a,b**); In the MEUS group, hepatocytes swelled, and erythrocytes accumulated in the sinusoids (**c**), the swollen hepatocytes deformed and compressed the sinusoids and perisinusoidal space. A large number of erythrocytes accumulated in the central veins (**d**), periportal connective tissue hemorrhaged (**e**). The targets showed map-like necrosis in the MEUS group after 48 h (**f**).

In the control groups, HE sections showed that the hepatic cord and plate structure remained intact after insonation. There were small numbers of erythrocytes scattered in the sinusoids which were of normal size. There were four kinds of lesions in the MEUS group after insonation: 1. hepatocytes were swollen and cloudy, compressing the sinusoids and perisinusoidal space at the wound surface; 2. a large number of erythrocytes that accumulated in the sinusoids, perisinusoidal and small veins, distant from the wound surface. 3. significant hemorrhage into periportal parenchyma. 4. scattered necrosis with infiltration of inflammatory cells appeared within the liver tissue in the insonation area. After 48 h, the surfaces of the wounds became gray and smooth and without any bleeding (bleed visual scores were grade 0). The HE sections showed that the area of the lesion was about 1.5 cm away from the wound surface, and the necrosis in the lesion area expanded. There was some degeneration surrounding the peripheral zone of the necrotic cells. Infiltration of a few inflammatory cells was seen in the areas of degenerated cells. There was a clear boundary between the lesions and normal tissues, and a small number of erythrocytes were found to be scattered in liver sinusoids near the lesions (Fig. 4).

## Discussion

The current study investigated four topics: (1) Creation a new trauma model - liver avulsion injury, whose type of injury was scale II [7], and characterized by a large number of steady bleeding sites. (2) Use of a new ultrasonic cavitation therapeutic device. (3) Analysis of the relationship between the bleeding rate and acoustic density (PI/AUC), and visual evaluation of bleeding. (4) Documentation of the range of tissue damaged by therapeutic cavitation after insonation.

Cavitation is a method by which non-thermal effects are used for temporary acoustic hemostasis. The results of the current study showed that when I_SPTA_ was less than 2.0 W/mm^2^ and the duty cycle was lower than 4%, mainly cavitation and not thermal effects occurred [24]. Angiotripsy [19], [20] is a technique in which extrinsic contrast agent microbubbles is injected intravenously to increase cavitation nuclei in the target tissue. The cavitation threshold is reduced, and the effect of transient cavitation is increased through damage to small blood vessels. The temperature during these experiments only increased by 3–5°C [25], or even declined by 4.1°C (from 31.1°C to 27°C) [20], which avoids thermal effects, structural deformation, and prevents damage to surrounding normal tissues. Moreover, the introduction of microbubbles can be used to monitor the bleeding in liver tissue during the process of hemostasis by therapeutic cavitation.

Microbubble-enhanced ultrasound has been shown to damage small arteries of mesenteric and auricular veins, resulting in structural damage to endothelial cells and the formation of thrombosis within the small arteries and veins [26], [27]. The use of pulsed focused ultrasound cavitation in our previous studies temporarily blocked the blood flow of rabbit livers by microbubbles [28]. We have found previously that microbubble-enhanced low intensity pulse non-focused ultrasound could interrupt the circulation of liver, spleen of rabbits [28]–[30], and splenic occlusion could last up to 1 hour when combined with prothrombin [30]. In addition, the non-enhanced area in the circulation of the liver after double-cavitation treatment was larger than that of single–cavitation treatment [31]. The high-pressure pulse focused ultrasound combined with microbubbles on the vessels of the left hepatic lobe. CEUS showed the area of the perfusion defect was about 2–3 cm [32]. Microbubble-enhanced and low-intensity ultrasound were able to completely block tumor perfusion [19]. In previous experiments, hemostasis was achieved in a trauma model consisting of neat cuts on the liver and spleen, indicating that MEUS significantly reduced or stopped bleeding within 3–4 mins of insonation[21], [22]. Forty-eight h later, after the liver had been replaced into the peritoneum, the target tissue showed necrosis by naked-eye [22]. In the current study, the MEUS group stopped bleeding almost completely after insonation, although slow oozing was still seen around the damaged large vessels. The overall flow from the wound decreased more than 10-fold. Histological examination showed that the hepatocytes were swollen and cloudy, compressing the sinusoids and perisinusoidal space at the wound surface. There were a large number of erythrocytes that accumulated in the sinusoids, perisinusoidal and small veins, distant from the wound surface. We believe that hemostatic effect was achieved as hepatocytes and capillary walls were subjected to various degrees of damage. Sinusoids and perisinusoidal spaces became compressed, and small vessels became congested in the portal areas of the bleeding site. Therefore, the blood flow was obstructed in the target blood vessels. The results suggested that the therapeutic cavitation produced by MEUS could achieve hemostasis, and could be applied to trauma of liver avulsion as well.

In the current study, CEUS showed that the PI and AUC of the MEUS group were significantly decreased compared with those of pre-insonation, indicating that the blood supply of the hepatic tissue was distinctly reduced in insonation region. The decrease in the bleeding rate after insonation positively correlated with the changes in PI and AUC. The reduction in the bleeding rates after insonation, and visual scores of bleeding, were also positively correlated. However, bleeding rates in the control groups after insonation were also lower than pre-insonation (Table 1). However the differences between pre-insonation and after insonation were not statistically significant (P>0.05). Therefore, it is necessary to deal with traumatic bleeding in a timely fashion. In addition, CEUS showed that the flow of large blood vessels in the insonation region could not be completely blocked by MEUS. The causes of this failure might be that ultrasonic therapeutic cavitation mainly breaks small blood vessels as evidenced by the small amount of oozing seen on the wound surface after insonation. However, the clinical avulsion injury usually involves tearing of the parenchyma and producing irregular damage to different sizes of vessels. The model used in this study produces a smooth surface which may be more amenable to treatment by MEUS than actual clinical conditions. Therefore, the clinical utility and parameters to optimize such treatment require further study.

This experiment almost completely blocked the circulation of hepatic blood using a therapeutic device producing pulsed non-focused ultrasound cavitation, and reducing the total amount of bleeding by at least ten-fold. In addition, the introduction of the microbubble contrast agent provided accurate information for monitoring and detecting the rupture of tiny blood vessels during the treatment. MEUS has the potential to become a useful new method for the clinical treatment of liver avulsion injury.

## Supporting Information

Checklist S1 **ARRIVE Checklist.**
(DOC)
